# Delving Into Retinoblastoma Genetics: Discovery of Novel Mutations and Their Clinical Impact: Retrospective Cohort Study

**DOI:** 10.1002/cam4.70922

**Published:** 2025-05-02

**Authors:** Mohammad Faranoush, Masood Naseripour, Pooya Faranoush, Zeinab Davoodi‐Moghaddam, Alireza Jahandideh, Negin Sadighnia, Delbar Daneshjou, Parisa Shams, Ahad Sedaghat, Reza Mirshahi, Shirin Ravanbod, Farzaneh Nasirnejad, Ali Elahinia, Davood Bashash

**Affiliations:** ^1^ Pediatric Growth and Development Research Center Institute of Endocrinology and Metabolism, Iran University of Medical Sciences Tehran Iran; ^2^ Eye Research Center The Five Senses Institute, Rassoul Akram Hospital, Iran University of Medical Sciences Tehran Iran; ^3^ Iranian Hemophilia and Thrombophilia Association (MAHTA) Tehran Iran; ^4^ Department of Hematology and Blood Banking School of Allied Medical Sciences, Shahid Beheshti University of Medical Sciences Tehran Iran; ^5^ Department of Clinical Science Faculty of Specialized Veterinary Sciences, Science and Research Branch, Islamic Azad University Tehran Iran; ^6^ Cell and Developmental Biology Department Faculty of Sciences and Advanced Technologies in Biology, University of Science and Culture, ACECR Tehran Iran

**Keywords:** enucleation, MLPA, mutations, RB1, retinoblastoma

## Abstract

**Background:**

Retinoblastoma (Rb) is a rare intraocular malignancy that originates in the retina of children under 5 years of age. Approximately one‐third of children diagnosed with retinoblastoma are associated with germline mutations in one of the *RB1* alleles. In this study, we aim to identify *RB1* mutations in retinoblastoma patients using Sanger sequencing in combination with multiplex ligation‐dependent probe amplification (MLPA).

**Method:**

The genomic DNA of 167 Rb patients was isolated from peripheral blood and their clinical information was extracted from medical records. The mutations in the *RB1* gene were identified through PCR sequencing. Negative results from the PCR sequencing were further analyzed using MLPA reactions.

**Results:**

*RB1* mutations were identified in 56 of the 167 (33.5%) patients. The common mutation types were frameshift mutations (*n* = 19), followed by nonsense (*n* = 20), splicing (*n* = 8), missense (*n* = 5), and whole exon deletion (*n* = 2). The overall survival rate was 98.2%, with an average follow‐up duration of 59 months. Moreover, germline *RB1* mutation's correlation with enucleation rates is less pronounced in unilateral cases (12.1%) compared to bilateral cases (65.5%). A total of 13 novel mutations have been identified, of which four are specifically associated with enucleation.

**Conclusion:**

This study provides a comprehensive analysis of *RB1* germline mutations in a group of cases with Rb, leading to the identification of 13 novel mutations in Rb patients at a referral center in Iran. We expect that our findings will yield valuable insights to inform the management and genetic counseling of Rb patients, as well as their relatives who are at a higher risk.

## Graphical abstract

A comprehensive outline of the study, detailing the process from DNA extraction to the evaluation of RB1 mutations in relation to clinical features and patient outcomes.

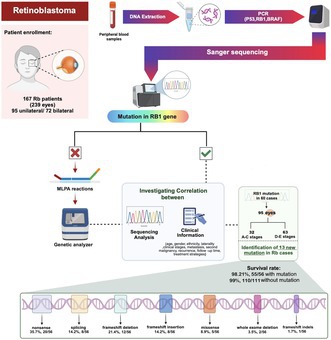

## 1 Introduction

Cancer is the leading cause of death worldwide, with its incidence rising steadily, resulting in a growing number of fatalities [1, 2, 3]. Retinoblastoma (Rb) is the most common intraocular tumor in children that originates in the retina, the tissue lining the Inner layer of the globe. While this condition is frequently diagnosed in children, it can also rarely manifest in adults [4, 5]. The worldwide occurrence of retinoblastoma remains steady, with an estimated incidence of one case per 15,000–20,000 live births, leading to around 9000 new cases annually. The disorder exhibits no validated geographic or population hotspots, but regions with high birth rates, particularly in Asia and Africa, bear the greatest disease burden [6]. The prevalence in these regions correlates with higher mortality rates, with 40%–70% of affected children in Asia and Africa succumbing to the disease compared to 3%–5% in Europe, Canada, and the United States [7].

Retinoblastoma is often initiated by a mutation in the RB1 gene, the first identified tumor‐suppressor gene [4, 8]. The constitutional loss of one RB1 allele predisposes individuals to cancer, and the loss of the other allele in a developing retinal cell triggers the formation of retinoblastoma tumors. This archetypal malignancy has revolutionized our understanding of cancer [9]. Biallelic loss‐of‐function mutations in the tumor suppressor gene RB1, situated on the chromosomal region 13q14, are responsible for initiating retinoblastoma in 95% of cases [10]. Retinoblastoma presents in two distinct forms: familial (heritable) and sporadic. The familial variant arises when individuals inherit a mutated copy of the Rb gene from one parent, indicating a hereditary predisposition. Conversely, the sporadic form is not inherited and may occur in any individual due to genetic alterations that accumulate during the first 2–5 years. Typically, heritable retinoblastoma appears at an earlier age and is most often multifocal or bilateral. In contrast, the majority of sporadic cases are unilateral, resulting from somatic mutations that affect both alleles of the RB1 gene [11]. In rare instances, sporadic retinoblastoma may develop without an RB1 mutation, resulting from somatic amplification of the MYCN gene [12]. Primary amplification of the MYCN oncogene is found in less than 2% of unilateral cases [13]. MYCN amplification can also occur in more typical retinoblastoma (RB) tumors where the RB1 gene is mutated (RB1−/−), resulting in the loss of functional RB protein. In these cases, the RB1 gene is not functional, leading to a loss of its tumor suppressor role [14]. The precise cellular origin of retinoblastoma remains unclear [15]. While live imaging data from early tumors in patients' eyes suggest the inner nuclear layer of the retina as the origin [16], experimental evidence suggests that human retinoblastoma may arise from differentiating cones. This is supported by findings indicating that retinoblastoma cells exhibit various elements of the cone precursor signaling circuitry, relying on it for their proliferation and survival [17], as well as the proliferation induced by RB1 knockdown in human cone precursors. Discrepancies in molecular, clinical, and histopathological aspects among RB1−/− tumors illustrate a progression characterized by a loss of differentiation and a reduction in the photoreceptor expression signature [18].

Unlike other types of cancer, retinoblastoma typically retains a wild‐type p53 tumor suppressor gene. However, its primary regulators, MDMX and MDM2, are often dysregulated. The general frequency of germinal RB1 gene mutation in patients with retinoblastoma is approximately 35% [19]. While p53 remains wild type in retinoblastoma, the dysregulation of its regulators (MDMX and MDM2) along with the loss of RB function contributes to a higher risk of secondary cancers in patients with hereditary retinoblastoma [20]. The relationship between BRAF mutations and RB in retinoblastoma is not as well documented as other genetic alterations like RB1 mutations or MYCN amplification. Though uncommon in retinoblastoma, the presence of both RB1 and BRAF mutations could theoretically result in a more aggressive tumor phenotype. BRAF mutations are not typically associated with primary retinoblastoma but could be relevant in the context of secondary cancers, particularly melanoma, in patients with hereditary retinoblastoma [21].

The treatment strategy for retinoblastoma depends on the timing of disease detection and socioeconomic factors as well as the infrastructure of the health system. In low‐ and middle‐income countries, the main goal of treatment is to save patients' lives through enucleation, with subsequent efforts directed towards salvaging one eye and vision. In several developing countries, delayed diagnosis from the initial clinical sign leads to a mortality rate of 70%. However, saving a life takes precedence over preserving vision, and the enucleation continues to be the main approach worldwide [22, 23]. Despite these challenges, advances in genome science and global communication have opened up new opportunities for providing a cure for children affected by retinoblastoma and enabled high‐risk families to access early detection resources to manage the condition effectively. Understanding the relationship between RB1 mutations and clinical presentation and outcomes is crucial for several reasons. Different types of mutations (e.g., missense, nonsense, frameshift) can have varying impacts on the function of the RB1 protein, leading to different clinical presentations and outcomes. This understanding can help in detecting prognostic values, improving patient care, and developing personalized treatment approaches. The development of molecular diagnostic techniques and next‐generation sequencing (NGS) has significantly enhanced the molecular screening of retinoblastoma. Recent advances in genomic science, particularly NGS technologies, have revolutionized genetic testing and counseling for retinoblastoma. The integration of NGS technologies into retinoblastoma genetic testing and counseling has transformed the landscape of ocular oncology. These advancements have not only improved the accuracy and speed of genetic analysis but also enhanced the ability to provide personalized treatment and comprehensive genetic counseling. As NGS technologies continue to evolve, they hold the promise of further improving patient care and outcomes in retinoblastoma and other genetic disorders [24]. Typically, RB1 gene testing involves analyzing the exons through direct sequencing and investigating copy number changes. In this study, we aimed to identify RB1 mutations in retinoblastoma patients using Sanger sequencing, supplemented by multiplex ligation‐dependent probe amplification (MLPA) for some patients. Due to sanctions in Iran, the NGS method was expensive and not available at that time. Additionally, we investigated the relationship between the RB1 mutations and clinical presentation and outcomes, hoping that our study provides new insights to guide the management and genetic counseling of Rb patients and their at‐risk relatives.

## 2 Methods

### 2.1 Patient Enrolment and Data Collection

A total of 168 patients diagnosed with retinoblastoma were enrolled at Rasool‐e‐Akram Hospital affiliated with Iran University of Medical Sciences, Tehran, Iran, between January 2017 and September 2023. The diagnosis was made according to standard ophthalmologic examination under anesthesia, clinical signs, and symptoms. All patients were diagnosed and treated based on the SIOP guidelines for retinoblastoma. Written informed consent was obtained from their parents before performing genetic tests. The essential information was retrieved retrospectively from medical records exclusively for eligible patients who had a confirmed diagnosis of Rb. The data extracted included demographic and clinical features like age at diagnosis, sex, ethnicity, laterality, clinical stages, metastasis, presence of second malignancy, recurrence, follow‐up time, and treatment strategies. The Rb grouping of patients was done according to the international classification of intraocular retinoblastoma (IIRC) [25]. One patient who failed to appear for further follow‐up was excluded from the study. Peripheral blood samples from the remaining 167 patients were stored at −20°C for subsequent DNA extraction. All the examinations were conducted with the ethical code of (IR.IUMS.REC.1398.1200) certified by the ethical committee of the Iran University of Medical Sciences.

### 2.2 Mutation Detection

#### 2.2.1 DNA Extraction

Genomic DNA was isolated from peripheral blood using the GeneElute kit according to the manufacturer's instructions.

#### 2.2.2 Polymerase Chain Reaction

To screen mutations of the RB1 gene, PCR was carried out using Taq 2X Master Mix (Biolabs, New England) and specific primers for mutations in the 26 coding exons and adjacent exon/intron regions of the RB1 gene. The amplification was then performed in a Mastercycler pro (Eppendorf, Germany). The cycling process was started with the denaturation step at 95°C for 2 min, followed by 35 cycles at 95°C for 30 s, annealing at the specific temperature for 30 s, 56°C for 30 s to 1 min, and a final extension step at 72°C for 5 min. In the next step, the melting curves of PCR reactions were closely monitored to ensure that a single PCR product was produced and there was no primer dimer.

#### 2.2.3 Sanger Sequencing and Mutation Annotation Process

##### 2.2.3.1 Sequencing

###### 2.2.3.1.1 Purified Products

The DNA samples were purified and then sequenced using the ABI 3130XL DNA analyzer (Applied Biosystems, Foster City, USA). This instrument is a high‐throughput genetic analyzer that uses capillary electrophoresis to separate and analyze DNA fragments.

###### 2.2.3.1.2 BigDye Terminator

The sequencing was performed using the BigDye terminator chemistry, which is known for its robust and flexible cycle sequencing capabilities. This method allows for accurate base calling and long read lengths, making it ideal for de novo sequencing and resequencing.

##### 2.2.3.2 Mutation Annotation

###### 2.2.3.2.1 Sequence Alignment

Each exon's sequence was aligned with the reference nucleotide sequence of the RB1 gene (GenBank L11910.1) using Sequence Scanner version 1.0 (Applied Biosystems, Streetsville, Ontario, Canada). This software enables the viewing, editing, printing, and exporting of sequence data, and generates graphical quality reports.

###### 2.2.3.2.2 Chromas

Chromas (version 2.6.5) was also used for sequence analysis. Chromas is a trace viewer that allows for the visualization and editing of chromatogram files, making it easier to identify and annotate mutations.

##### 2.2.3.3 Additional Information

###### 2.2.3.3.1 LOVD v.3.0

Additional information about RB1 gene mutations was obtained from the Leiden Open Variation Database (LOVD) version 3.0. LOVD is an open‐source DNA variation database that provides a comprehensive collection and display of genetic variants, facilitating the annotation and interpretation of mutations (http://www.lovd.nl/3.0/home) and COSMIC v100 (2024) (https://cancer.sanger.ac.uk/cosmic).

#### 2.2.4 Multiplex Ligation Probe‐Dependent Amplification

The SALSA MLPA (Multiplex Ligation‐dependent Probe Amplification) kit is a widely used tool for detecting copy number variations and point mutations in DNA. The limit of detection (LOD) for the SALSA MLPA kit refers to the smallest amount of DNA or the smallest change in DNA copy number that can be reliably detected by the assay. MLPA cannot detect any changes that lie outside the target sequence of the probes and will not detect copy number neutral inversion or translocations. Negative cases are from the PCR sequencing candidates in MLPA reactions. The SALSA MLPA probemix PO47‐B1 RB1 (MRC Holland) was used to detect single‐ to multiexon deletions or duplications at the RB1 locus. The probemix contains probes for detecting 26 of 27 RB1 exons. The procedure was performed according to the manufacturer's instructions. PCR amplicons ranging between 100 and 500 nt were separated on Genetic Analyzer 3500 (Applied Biosystem).

### 2.3 Statistical Analysis

Categorical and continuous variables were defined using percentages and mean ± SD, respectively. The differences between continuous variables were compared using Student's *t*‐test, while nonparametric Mann–Whitney *U* test and chi‐square or Fisher's exact tests were used to compare categorical variables. *p* ≤ 0.05 was considered statistically significant. All data were analyzed using the statistical software SPSS (version 16.0). Graphs were plotted by Graph Pad Prism software (version 8.0.2) and sigmaplot 14 (Systat Software).

## 3 Results

### 3.1 Clinical Characteristics

A total of 167 patients with Retinoblastoma were enrolled in this study. The patients were almost evenly divided by sex, with 87 male (52%) and 80 female (47.9%). The age at diagnosis ranged from 1 to 148 months, with an average age of 30.53 ± 22.16 months. It is worth noting that around 89.8% of retinoblastoma cases were diagnosed before the age of five. Among all Rb patients, 95 had unilateral and 72 had bilateral involvement. Unilaterally affected patients had an average age of 35.94 ± 24.6 months at the time of diagnosis, while bilateral cases had an average age of 23.4 ± 16 months. The majority of patients were Persian (55%), followed by Turk (15.5%), Kurd (6.5%), Arab (6.5%), and Lur (5.3%). Notably, all patients diagnosed with Rb, except for three cases, are alive. However, over half of them experienced advanced stages E and D of the tumor. Table 1 provides a summary of the demographic and clinical characteristics of these patients. Accordingly, RB1 mutations were detected in 56 cases, out of which 31 were female and 25 were male. These patients had a significantly younger onset age (24.71 ± 15.06 months) compared to those without the deficiency (33.47 ± 24.53 months, *p* = 0.013), with a higher percentage of bilateral cases (69.6% vs. 30.3%, *p* = 4.894e‐06). Nevertheless, there were no statistically significant differences in terms of metastasis, recurrence, sex, and clinical stages.

**TABLE 1:** Demographic and clinical characteristics of RB patients.

Characteristics	Total (*n* = 167)	RB1 mutated (*n* = 56)	RB1 wildtype (*n* = 111)	*p*
Age at diagnosis (month)				
Mean ± SD	30.53 ± 22.16	24.71 ± 15.06	33.47 ± 24.53	0.01395
Sex				
Female	80 (47.9%)	31 (55.3%)	49 (44.1%)	0.1977
Male	87 (52%)	25 (44.6%)	62 (55.85%)	
Ethnicities				
Persian	92 (55%)	27 (48.2%)	64 (57.6%)	0.4204
Turk	26 (15.5%)	11 (18.3%)	15 (14%)	0.5077
Kurd	11 (6.5%)	4 (6.6%)	7 (6.5%)	1
Arab	11 (6.5%)	4 (6.6%)	7 (6.5%)	1
Lur	9 (5.3%)	4 (6.6%)	5 (4.6%)	0.2851
Baloch	6 (3.5%)	1 (1.6%)	5 (4.6%)	0.4211
Turkmen	3 (1.7%)	1 (1.6%)	2 (1.8%)	1
Ozbek	1 (0.5%)	0 (0%)	1 (0.9%)	1
Afghan	2 (1.1%)	0 (0%)	2 (1.8%)	0.5368
NA	3 (1.7%)	0 (0%)	3 (2.8%)	
Laterality				
Unilateral	95 (56.8%)	17 (30.3%)	78 (70.2%)	4.894e‐06
Bilateral	72 (43.1%)	39 (69.6%)	33 (29.7%)	
Vital status				
Alive	164 (98.2%)	55 (98.2%)	109 (98.1%)	1
Dead	3 (1.79%)	1 (1.6%)	2 (1.86%)	
Recurrence				
With recurrence	115 (68.8%)	41 (73.2%)	74 (66.6%)	0.3876
Without recurrence	52 (31.1%)	15 (26.7%)	37 (33.3%)	
Secondary cancer				
Present	3 (1.79%)	2 (3.3%)	1 (0.9%)	0.2933
Absent	164 (98.2%)	54 (96.4%)	110 (99%)	
Metastasis				
Present	6 (3.5%)	3 (5%)	3 (2.8%)	0.668
Absent	161 (96.4%)	53 (94.6%)	108 (97.2%)	

International classification	(*n* = 239 Eyes)	(*n* = 95 Eyes)	(*n* = 144 Eyes)	*p*
A stage	5 (2%)	1 (1%)	4 (2.8%)	0.4031
B stage	22 (9.2%)	5 (5%)	17 (12.2%)	0.0699
C stage	57 (23.8%)	26 (27.3%)	31 (21.5%)	0.2205
D stage	24 (10%)	11 (11%)	13 (9.3%)	0.6701
E stage	131 (54.8%)	52 (54.7%)	79 (54.8%)	1

### 3.2 RB1 Mutations

**FIGURE 1:**
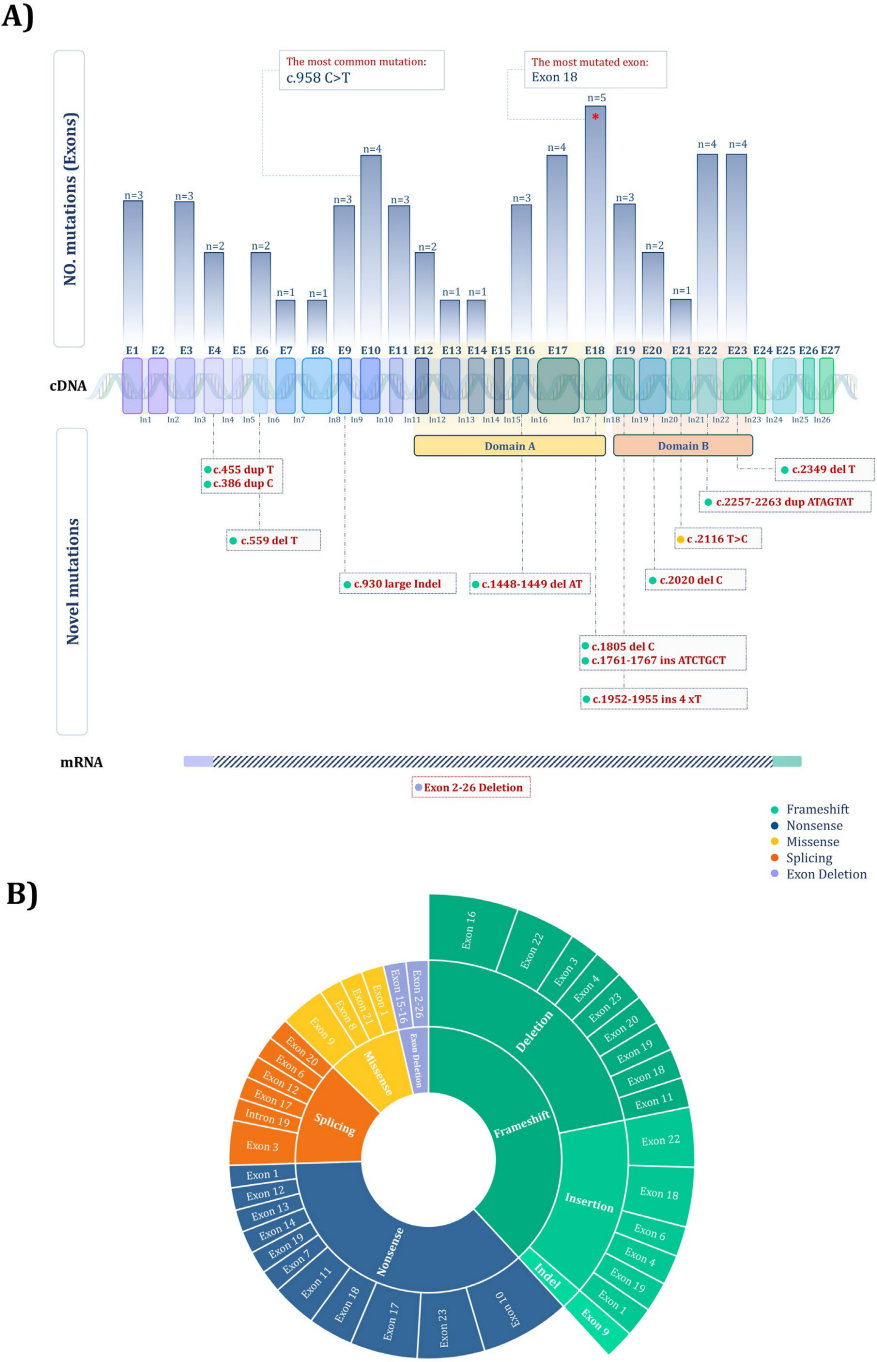
(A) Distribution of mutations detected in Rb patients across the RB1 gene. (B) Distributions of mutations in RB1 based on type.

Sequencing analysis of the DNA samples revealed RB1 mutation in 56 (33.5%) of 167 cases in this study (Table 2). All RB1 mutations were detected by sequencing, except for three mutations, which were identified using the MLPA method. These mutations include RB1 (deletion: exon 15–16), RB4 (deletion: exon 2–26), and RB12 (duplication: exon 18). Of these mutations, 85.7% (48/56) occurred within the exon, while 14.2% (8/56) were displayed in splicing sites. A schematic representation of detected germline mutations in the RB1 gene is provided in Figure 1A. The most common mutation type was nonsense mutations (35.7%, 20/56), followed by frameshift deletion (21.4%, 12/56), frameshift insertion (14.2%, 8/56), splicing (14.2%, 8/56), missense (8.9%, 5/56), whole exon deletion (3.5%, 2/56), and a frameshift Indel mutation (1.7%, 1/56). Figure 1B shows a better overview of the detected alterations based on mutation type. Among the exons, exon 18 carried the most mutation frequency and was found in 5 patients. Besides that, mutations were located in exons 10, 17, 22, and 23 also reported in 4 patients. However, no mutations were seen in exons 5, 24, 25 (Figure 1A). Noteworthy, more than half of the detected mutations (30/56) were found at regions encoding two conserved domains A (exon 12–18) and B (exon 19–23) of pRB pocket including 12/20 nonsense mutations, 9/12 frameshift deletion, 3/8 splicing mutations, 5/8 frameshift insertions mutations, and 1/5 missense mutations. The mutation rate was equal between the two domains, with 28% (16/56) and 25% (14/56) respectively in domains A and B. After conducting a parental origin analysis, it was found that 4/30 cases were familial while 26/30 cases were sporadic. Out of 20 patients with nonsense mutations, 15 patients (75%) had C to T transitions in CGA codons in exons 10, 11, 12, 14, 17, 18, and 23. This alteration in exon 10 (c.958 C>T) is the most prevalent mutation among Rb patients in the current study which was identified in 4 bilateral patients. Considering coexistent TP53 and BRAF mutations, only RB‐68 had c.1789 C>G alteration in BRAF.

**TABLE 2:** A summary of RB1 germline mutations in the 56 Rb patients.

IDs	Location	Change in cDNA	Change in protein	Mutational type	Het/Hom	Father	Mother	Laterality	Novel/reported
RB‐51	Exon 1	c.66–89 dup 23 bp	p.pro27 > *R*	Insertion	HETERO	HETERO	N	Uni	Reported
RB‐6	Exon 1	c.22 A>T AAA>TAA	p.(Lys8*)	Nonsense	HETERO	HETERO	N	Uni	Reported
RB‐86	Exon 1	c.59 C>T	p.(Pro20Leu)	Missense	HETERO	HETERO	N	Uni	Reported
RB‐143	Exon 3	c.380 + 10 C>G		Splicing	HETERO	HETERO	N	Bi	Reported
RB‐133	Exon 3	c.289 del G	p.(Glu97Asnfs*14)	Deletion	HETERO	N	N	Bi	Reported
RB‐8	Exon 3	c.380 + 1 G>T		Splicing	HETERO	N	N	Bi	Reported
RB‐25	Exon 4	c.385 dup C	p.(His129Profs*1)	Insertion	HETERO	N	N	Bi	Novel
RB‐10	Exon 4	c.455 dup T	p.(Leu152Phefs*5)	Insertion	HETERO	HETERO	N	Bi	Novel
RB‐19	Exon 6	c.607 + 1 G>A		Splicing	HETERO	N	N	Uni	Reported
RB‐58	Exon 6	c.559 del T	p.(Ser187Leufs*5)	Deletion	HETERO	N	N	Bi	Novel
RB‐20	Exon 7	c.610 G>T	p.(Glu204*)	Nonsense	HETERO	N	N	Bi	Reported
RB‐164	Exon 8	c.857 A>G	p.(Asp286Gly)	Missense	HETERO	N	HETERO	Uni	Reported
RB‐17	Exon 9	c.920 C>T ACA>ATA	p.(Thr307Ile)	Missense	HETERO	—	—	Uni	Reported
RB‐27	Exon 9	c.920 C>T ACA>ATA	p.(Thr307Ile)	Missense	HETERO	N	HETERO	Bi	Reported
RB‐106	Exon 9	c.930 large Indel Truncated RB1		Indel	HETERO	N	N	Uni	Novel
RB‐59	Exon 10	c.958 C>T CGA>TGA	p.(Arg320*)	Nonsense	HETERO	N	N	Bi	Reported
RB‐61	Exon 10	c.958 C>T CGA>TGA	p.(Arg320*)	Nonsense	HETERO	N	N	Bi	Reported
RB‐89	Exon 10	c.958 C>T CGA>TGA	p.(Arg320*)	Nonsense	HETERO	N	N	Bi	Reported
RB‐163	Exon 10	c.958 C>T CGA>TGA	p.(Arg320*)	Nonsense	HETERO	N	N	Bi	Reported
RB‐102	Exon 11	c.1063–1064 del AG	p.(Arg355Asnfs*6)	Deletion	HETERO	N	HETERO	Bi	Reported
RB‐128	Exon 11	c.1072 C > T CGA>TGA	p.(Arg358*)	Nonsense	HETERO	N.A.	HETERO	Bi	Reported
RB‐18	Exon 11	c.1072 C > T CGA>TGA	p.(Arg358*)	Nonsense	HETERO	N	N	Bi	Reported
RB‐138	Exon 12	c.1128–2 A>G		Splicing	HETERO	N	N	Bi	Reported
RB‐125	Exon 12	c.1147 C>T	p.(Gln383*)	Nonsense	HETERO	N	N	Bi	Reported
RB‐39	Exon 13	c.1279A>T	p.(Lys427*)	Nonsense	HETERO	N	N	Bi	Reported
RB‐22	Exon 14	c.1363 C>T CGA>TGA	p.(Arg455*)	Nonsense	HETERO	N	N	Bi	Reported
RB‐62	Exon 16	c.1448–1449 del AT	p.(His483fs*8)	Deletion	HETERO	N	N	Bi	Novel
RB‐110	Exon 16	c.1488 del	p.(Thr497Hisfs*22)	Deletion	HETERO	N	N	Bi	Reported
RB‐92	Exon 16	c.1494 del T	p.(Tyr498*)	Deletion	HETERO	N	N.A.	Bi	Reported
RB‐68	Exon 17	c.1654 C>T	p.(Arg552*)	Nonsense	HETERO	N	N	Uni	Reported
RB‐15	Exon 17	c.1654 C>T	p.(Arg552*)	Nonsense	HETERO	N	N	Bi	Reported
RB‐166	Exon 17	c.1654 C>T	p.(Arg552*)	Nonsense	HETERO	N	N	Bi	Reported
RB‐162	Exon 17	c.1499–2 A>G		Splicing	HETERO	N	N	Bi	Reported
RB‐71	Exon 18	c.1666 C>T	p.(Arg556*)	Nonsense	HETERO	N	N	Bi	Reported
RB‐11	Exon 18	c.1805 del C	p.(Arg563Glnfs*9)	Deletion	HETERO	N	N	Bi	Novel
RB‐119	Exon 18	c.1759 G>T	p.(Glu587*)	Nonsense	HETERO	HETERO	N	Bi	Reported
RB‐45	Exon 18	c.1761–1767 ins ATCTGCT C GAA>GAT	p.(Glu587Aspfs*21)	Insertion	HETERO	N	N	Bi	Novel
RB‐12	Exon 18	E = 18 Duplication		Insertion	—	—	—	Uni	Reported
RB‐131	Exon 19	c.1952–1955 ins 4 xT		Insertion	HETERO	N	N	Bi	Novel
RB‐30	Exon 19	c.1954 A>T AAA>TAA	p.(Lys652*)	Nonsense	HETERO	N	N	Bi	Reported
RB‐37	Exon 19	c.887–888 del GA GAG>GAC	p.(Glu629Aspfs*22)	Deletion	HETERO	N	N	Bi	Reported
RB‐40	Intron 19	c.1960 + 1 del G		Splicing	HETERO	N	N	Bi	Reported
RB‐57	Exon 20	c.2106 + 2–2106 + 5 del TAAG		Splicing	HETERO	N	N	Bi	Reported
RB‐9	Exon 20	c.2020 del C	p.(Pro674Glnfs*2)	Deletion	HETERO	N.A.	HETERO	Bi	Novel
RB‐77	Exon 21	c.2116 T>C	p.(Cyc706Arg)	Missense	HETERO	N	N	Bi	Novel
RB‐87	Exon 22	c.2261 dup T	p.(Phe755Ile +2Fs*)	Insertion	HETERO	N	N	Bi	Reported
RB‐115	Exon 22	c.2240 del A	p.(Glu747Gly +65*)	Deletion	HETERO	HETERO	N	Bi	Reported
RB‐117	Exon 22	c.2228 del T	p.(Leu743*)	Deletion	HETERO	N	N	Bi	Reported
RB‐48	Exon 22	c.2257–2263 dup ATAGTAT	p.(Phe755leufs*2)	Insertion	HETERO	N	N	Uni	Novel
RB‐34	Exon 23	c.2349 del T	p.(His784Thrfs*26)	Deletion	HETERO	N	N	Bi	Novel
RB‐66	Exon 23	c.2359 C>T	p.(Arg787*)	Nonsense	HETERO	N	N	Bi	Reported
RB‐69	Exon 23	c.2359 C>T	p.(Arg787*)	Nonsense	HETERO	N	N	Bi	Reported
RB‐85	Exon 23	c.2359 C>T	p.(Arg787*)	Nonsense	HETERO	N	N	Bi	Reported
RB‐64	Intron 24	c.2520 + 2 T>C		Splicing	HETERO	N	N	Uni	Reported
RB‐4		Deletion = 2–26		Exon Deletion	HETERO	HETERO	N.A.	Bi	Novel
RB‐1		Deletion = 15–16		Exon Deletion	HETERO	N	N	Bi	Reported

Abbreviations: N, Normal; N.A., Not available.

The study found that 48 distinct mutations in the RB1 gene caused retinoblastoma in 56 patients. The mutations were detected in 54.1% of patients with bilateral (39/72) and 17.8% of patients with unilateral retinoblastoma (17/95) (Table 1). As mentioned, the germline mutation rate was significantly higher in patients affected bilaterally than in those affected unilaterally (*p* = 4.894e‐06). In total, 95 eyes underwent germline mutation in this study, of which 32 had A‐C stages and 63 experienced higher stages D‐E. No significant difference in metastasis, sex, and clinical stage at diagnosis was observed between the groups with and without mutations. The mean age at diagnosis for patients with bilateral retinoblastoma, differentiated by the presence or absence of germline RB1 mutations, was observed to be 22.9 months (±13.1 months) and 24.3 months (±19.2 months), respectively. In contrast, unilateral cases presented with mean ages of 28.8 months (±18.5 months) for those with germline RB1 mutations and 37.5 months (±25.5 months) for those without such mutations.

#### 3.2.1 Novel RB1 Mutations

**FIGURE 2:**
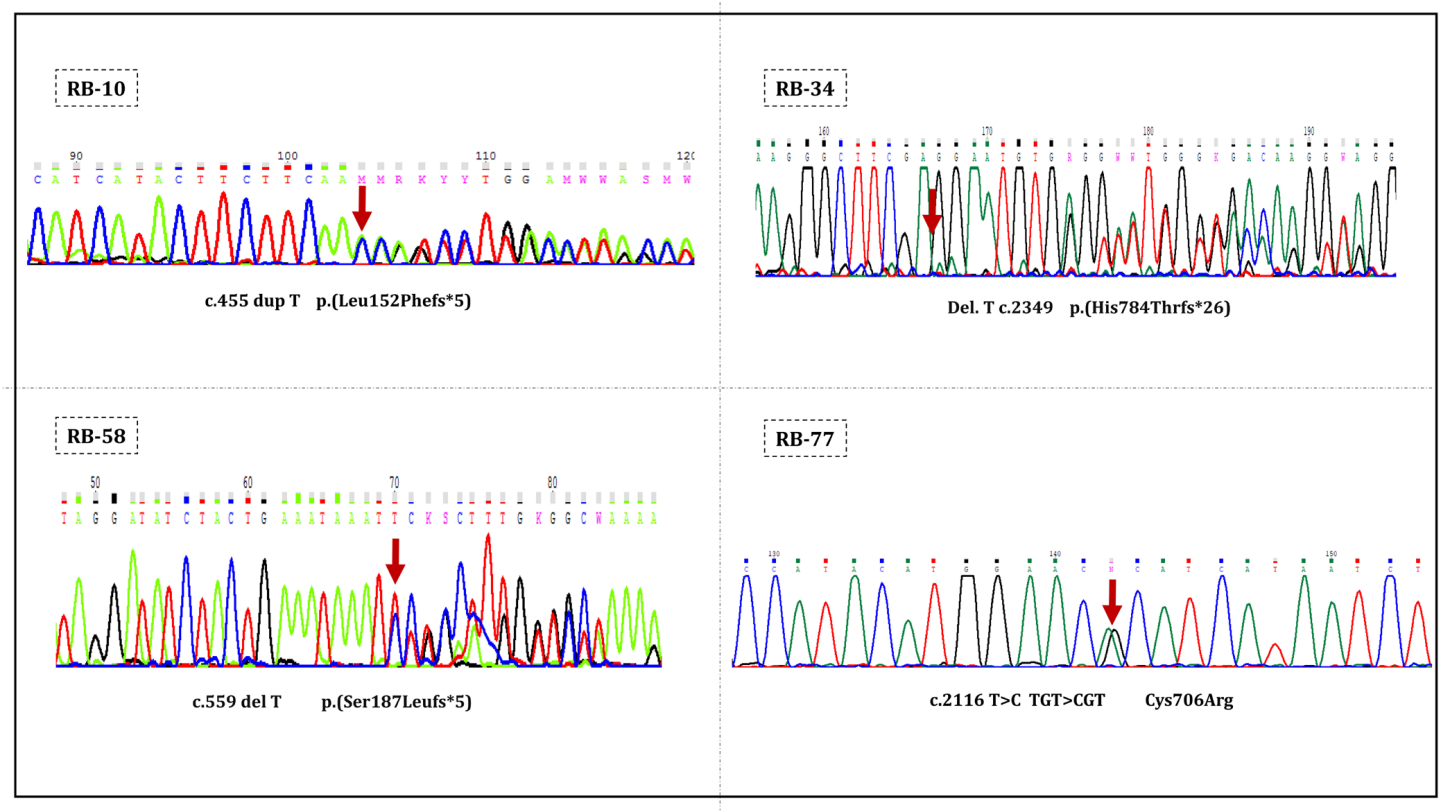
Sequencing analysis of the four novel RB1 gene mutations associated with enucleation (the reverse sequence was represented for RB‐10 and RB‐58).

We have identified 13 new mutations in our Rb cases, and their details can be found in Table 2 and Figure 1A. Out of these mutations, there were 11 frameshift mutations, 1 missense, and 1 whole exon deletion. Five insertions, five deletions, and one Indel were detected in the RB1 gene as novel frameshift alterations. Deletions and insertions caused premature stop codons in the reading frame, leading to the loss of coding exons and a truncated RB protein. Of all novel frameshift deletions, two were located in the protein‐binding sites of the pRB pocket's domain A (2/5), whereas two were in domain B (2/5). Considering parental origin, the deletion of a C nucleotide at positions 2020 of coding exon 20 (c.2020delC) was inherited from RB‐9's mother, and one insertion of T nucleotide in exons 4 was also inherited from the fathers of RB‐10 patient. In addition, a novel duplication ATAGTAT in locations 2257 to 2263 of the exon 22 at regions encoding domain B caused a change in the RB1 gene. It is worth mentioning that we discovered a large Indel in exon 9 of patient RB‐106. In the current study, a deletion from exons 2 to 26 of the RB1 gene was detected in a patient with stage E Rb (RB‐4) using MLPA. This deletion was inherited from the patient's father. Of note, a substitution of T to C has been determined in exon 21, leading to a missense mutation in coding domain B. The sequencing analysis of the 13 new variants is shown in Figure 2 and Figure S1.

### 3.3 Prognosis of Rb Patients

The median follow‐up time of Rb patients was 59 months (53–57 months). While 164 cases still survived, only 3 of them died. Therefore, the overall survival (OS) rate was calculated as 98.2% (164/167). As represented in Table 3, Rb patients with unilateral involvement had a higher OS rate than those with bilateral involvement in this cohort. In addition, the presence or absence of mutation did not have a substantial effect on the survival of patients in this study, with a survival rate of 98.21% (55/56) and 99% (110/111) for cases with mutation and those without mutation, respectively. During the follow‐up period, 115 patients with Rb experienced recurrence. Out of these, 112 patients survived, giving an overall survival rate of 97.3%. Three patients died and 90 patients discontinued treatment.

**TABLE 3:** Enucleation and survival of Rb patients.

Groups	Survival		Enucleation	
	*n*	Rate	*n*	Rate
Laterality				
Unilateral (*n* = 95)	95	100%	41	43.10%
Bilateral (*n* = 72)	69	95.80%	29	40.20%
RB1 Mutation				
With mutation (*n* = 56)	55	98.21%	24	42.85%
Without mutation (*n* = 111)	110	99%	46	41.44%
Recurrence				
With recurrence (*n* = 115)	112	97.30%	48	41.70%
Without recurrence (*n* = 52)	52	100%	22	42.30%
Treatment				
Intra‐arterial chemotherapy (*n* = 83)	83	100%	33	39.70%
Systemic chemotherapy (*n* = 49)	46	93.80%	21	42.80%
Intra‐arterial and Systemic chemotherapy (*n* = 30)	30	100%	14	46.60%
Only Local (*n* = 3)	3	100%	0	0%
Only Enucleation (*n* = 2)	2	100%		

Among the 167 patients considered, 70 (41.9%) underwent eye enucleation. Of these, 68 cases (41.9%, 68/162) received chemotherapy before the enucleation procedure and obtained an OS rate of 95.5% (65/68). The enucleation rates observed in patients who received treatment varied based on the type of therapy administered. Specifically, the rates were 39.7% for those who underwent intra‐arterial chemotherapy (IAC), 42.8% for patients receiving systemic chemotherapy, and 46.6% for those who received a combination of both treatments. This indicates a trend where the combination therapy yields a higher enucleation rate compared to either treatment alone. Further investigation may be needed to fully understand the implications of these findings in clinical practice. On the other hand, 97 cases of all patients (58%, 97/167) did not require enucleation and had a 100% OS rate (97/97). The investigation of 56 cases of Rb with mutations revealed that the enucleation rate was 42.85% (24/56), while the survival rate was 95.8% (23/24). The presence of a germline mutation in the RB1 gene is associated with a significantly higher enucleation rate in bilateral cases, recorded at 65.5% (19/29). In contrast, this rate drops to 34.4% (10/29) when the mutation is absent. Furthermore, in unilateral cases, the enucleation rates reflect 12.1% (5/41) for those with mutations, compared to 87.8% (36/41) for those without. These findings are consistent with the results of prior studies, reinforcing the established correlation between RB1 mutations and enucleation outcomes, and vary significantly between unilateral and bilateral cases [26, 27]. Among the different types of mutations, missense mutations with three enucleation reports showed the highest enucleation rate of 60% (3/5), followed by nonsense (40%, 8/20), splicing (37.5%,3/8), and frameshift mutations (33%, 7/21). Moreover, four novel detected mutations in this study including c.2349 del T (p.His784Thrfs*26) in exon 23, c.455 dup T in exon 4 (p.Leu152Phefs*5), c.559 del T (p.Ser187Leufs*5) in exon 6, c.2116 T>C (p. Cyc706Arg) in exon 21 may relate to the enucleation. Patients without RB1 mutations also showed a high enucleation rate of 41.44% (46/111), but their OS rate was still impressive with 95.6% (44/46) survival.

Notably, the coexistence of BRAF and RB1 mutations in RB‐68 may have resulted in stage E retinoblastoma and the patient underwent enucleation. Further research is needed to explore the relationship between these two mutations.

## 4 Discussion

The RB1 gene, an essential tumor suppressor gene, was the first to be molecularly characterized and is expressed in several tissues [28]. Approximately one‐third of children diagnosed with retinoblastoma are associated with germline mutations in one of the RB1 alleles [29]. These mutations are present in all cells of the body, and detecting them is critical for both clinical management and precise genetic counseling. As far as we know, the current study dealt with the most extensive cohort of Iranian patients with Rb whose germline mutations in the RB1 gene have been comprehensively analyzed. Among 167 Rb patients in this study, 33.5% (56/167) had a germline mutation in the RB1 gene, implying that the mutation detection rate is lower than the previously reported 49% (52/106) detection in the Iranian population [30]. Moreover, our study has reported a lower rate of RB1 germline mutation compared to studies conducted in other countries [31, 32]. It is important to note that the mutation detection rate in blood depends on the number of patients with germline mutations, and this proportion varies depending on each group of Rb patients studied. This variability may contribute to the observed differences in detection rates and highlights the need for further research to understand the genetic landscape of retinoblastoma in various populations [26]. This variability in mutation detection rate might be justified by the different techniques employed, sample sizes recruited in these studies, and geographical or racial diversity [33]. Several factors could contribute to the lower detection rate of RB1 mutations, including: technical limitations leading to missed mosaic variants or deep intronic mutations by conventional sequencing techniques, incomplete coverage of all regions of the RB1 gene in the analysis, and low‐level mosaicism in some patients that is difficult to detect. Some patients in the without mutation group may, in fact, carry undetected mutations. To address this issue, it is essential to use more comprehensive and sensitive detection methods, such as NGS, which can identify a broader range of mutations with higher accuracy [33].

Until now, over 1900 different mutations in the RB1 gene have been reported across the world [34, 35], and they are widely dispersed throughout the gene. However, the mutations discovered in this study were primarily concentrated in the highly conserved pocket region, domains A and B (Figure 1A). These domains interact with E2F transcription factors and suppress their activity. Since this factor is responsible for regulating the G1 to S phase transition of the cell cycle, mutations in the RB1 gene can impact the structure and function of pRB, increasing cell proliferation [36, 37]. Several studies detected mutations affecting the A and B domains not only in sporadic cancers [38, 39], but also as germline mutations in patients with retinoblastoma [40, 41]. Shahraki et al. indicated that 52% of Iranian Rb patients had an alteration of these domain‐relevant exons, and exon 19 was the most commonly affected [30]. Although there is no definite hot spot for RB1 mutations, RB1 exon 18 is repeatedly mutated in our study and may be a key pathogenic mutation in this population.

We found only wild BRAF mutations in our patients. Research continues to explore the complex genetic landscape of cancers, and while BRAF mutations are not a primary driver in retinoblastoma, understanding their role in secondary malignancies could be significant for comprehensive patient care. Mutations in BRAF, especially the V600E mutation, are common in various types of cancer, including melanoma, colorectal cancer, and thyroid cancer [42].

We did not observe any simultaneous mutations in p53 and RB in our patients. This finding indicates that retinoblastoma does not exhibit the coexistence of these mutations. The inactivation of the p53 pathway in retinoblastoma suggests that this cancer does not originate from cells that are inherently resistant to programmed cell death, which contradicts previous assumptions. Additionally, the MDM2 oncogene is intricately regulated by MYCN and plays a pivotal role in the development of retinal cancer through the degradation of p53, thereby facilitating tumor progression. This underscores the potential of targeting both MYCN and MDM2 as a novel therapeutic strategy. Inhibiting MDM2 may restore p53 function, which could enhance treatment outcomes significantly [43].

Some evidence revealed that specific types of mutations can influence the manifestation of the disease and its response to treatment [44, 45]. Particularly, Nonsense and frameshift mutations often result in multifocal bilateral tumors [46], whereas intronic splice mutations may lead to less severe forms of the disease with a reduced risk of tumor progression [47, 48]. In this study, the most common mutation types were nonsense (35.7%, 20/56), followed by frameshift deletion (21.4%, 12/56), frameshift insertion (14.2%, 8/56), and splicing (14.2%, 8/56). Importantly, 95% of patients with nonsense mutations (19/20), and 71.4% of patients with frameshift (15/21) expressed bilateral involvement. These findings are in concordance with previous studies [31, 49]. In addition, total germline mutations were significantly higher in bilateral than unilateral patients (69.6% vs. 30.3%, *p* = 4.894e‐06). As some unilateral Rb patients may also have germline RB1 mutations and are at risk of other malignancies, such as sarcoma [50], genetic testing should be applied to all involved individuals.

Our study presents intriguing deviations from previously established patterns in the literature regarding mutation types and their clinical implications. According to existing literature, missense mutations are more commonly observed in unilateral cases, which are often diagnosed later than bilateral cases. This delay in diagnosis has been associated with a higher enucleation rate, suggesting that the disease progresses further in these patients before intervention [50].

However, our findings reveal a different trend. Importantly, we observed that 95% of patients with nonsense mutations (19 of 20) and 71.4% of patients with frameshift mutations (15 of 21) exhibited bilateral involvement. This significant prevalence of bilateral disease in patients with nonsense and frameshift mutations contrasts with the higher incidence of unilateral involvement typically seen with missense mutations.

The higher rate of bilateral involvement in patients with nonsense and frameshift mutations may indicate a more aggressive phenotype associated with these mutation types. These mutations likely result in a more severe functional loss of key proteins, leading to a broader manifestation of the disease. This suggests that while missense mutations may allow for some residual protein function, nonsense and frameshift mutations result in a complete loss of function, thereby contributing to the bilateral presentation [51, 52].

Our study indicated an overall survival rate exceeding 98.2%, with an average follow‐up duration of 59 months. This is notably higher than the 91.2% 3‐year survival rate for children in upper‐middle‐income countries, as reported by the Global Retinoblastoma Outcome Study group in 2022 [53]. Besides, we found that the enucleation rate was similar among patients with and without RB1 mutations. The time of diagnosis significantly impacts both globe and overall survival rates in retinoblastoma. Diagnosis of disease at an early stage enables timely intervention, which can help preserve vision in affected eyes [54, 55]. Noteworthy, among the different types of mutations, missense mutations were associated with the highest enucleation rate. This finding further strengthens the idea that the specific type of genetic mutation can significantly impact treatment approaches and subsequent outcomes. However, the relationship between genotype and phenotype of retinoblastoma is intricate, and various factors can influence its understanding. Thus, further in‐depth investigation is required to comprehend the matter completely.

The present study also identified 13 novel mutations mostly displaying frameshift, which is associated with high penetrance (> 90%) and bilateral Rb [56]. Accordingly, except for the RB‐48 mutation, all retinoblastoma patients with novel frameshift mutations showed bilateral involvement. It is worth noting that four novel identified mutations in this study are related to enucleation (Figure 2). Among them, the alteration of the RB1 open reading frame in exons 4, 23, and 6 of RB‐10, ‐34, and ‐58 patients exacerbated the condition and progressed rapidly to stages D and E. Identification of the spectrum of RB1 mutations could help in the development of better‐targeted therapies and provide insight into the disease management of the relatives at risk. Nowadays, RB1 genetic testing is widely used to screen and detect carriers of RB1 mutations among relatives of affected individuals, as well as for prenatal diagnosis. These tests facilitate the clinical management of retinoblastoma, improve visual outcomes in affected children, and reduce noncarriers of at‐risk relatives from excessive anesthesia and aggressive screening. In some cases of bilateral retinoblastoma, detecting any mutations may not be possible. This could be because of a retinal tag in one eye or limitations in laboratory techniques. Notably, Sanger sequencing primarily focuses on exonic regions, which can result in the omission of pathogenic mutations located in intronic areas that may affect splicing [57]. Moreover, this method might not fully capture all variants, particularly complex rearrangements or large deletions [58]. Conversely, MLPA is effective at detecting known copy number variations, yet it has limitations in identifying novel mutations or those outside the designated targeted regions [59]. MLPA may produce false negatives for mutations that do not influence copy number, such as point mutations or small insertions and deletions [26]. A multifaceted approach in genetic testing and advanced laboratory methods such as NGS may be necessary to improve sensitivity and identify such mutations accurately.

Our study underscores the importance of genetic profiling in predicting disease presentation and tailoring treatment strategies. By identifying patients with high‐risk mutations, such as nonsense and frameshift mutations, we can prioritize early and aggressive therapeutic interventions to mitigate the severity of the disease.

These findings highlight the need for further research to explore the molecular mechanisms driving these differences and to develop targeted therapies that address the specific challenges posed by various mutation types. Our study contributes to a more nuanced understanding of the genetic basis of disease presentation and reinforces the value of personalized medicine in managing complex conditions.

## 5 Conclusion

**FIGURE 3:**
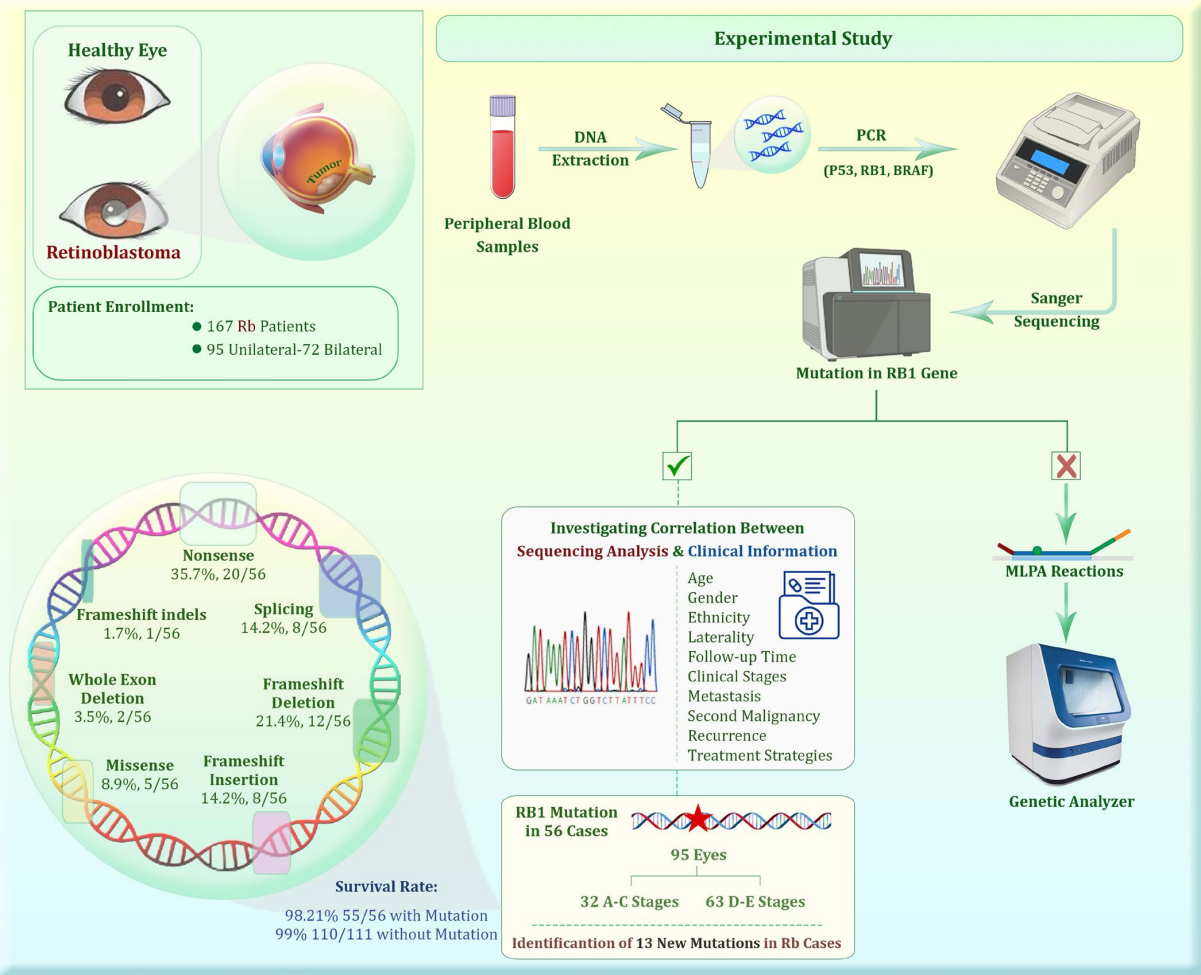
An overview of the study from DNA extraction to investigating the correlation between RB1 mutations and clinical and outcome information.

Although the etiology of retinoblastoma is well understood and advances in its management have improved patients' survival rates and globe preservation, obvious survival disparities remain between developed and developing countries. This is mainly due to a lack of awareness among the public and healthcare providers, as well as large cultural and socioeconomic differences, and variations in healthcare infrastructure among countries. Here, we present a comprehensive analysis of RB1 germline mutations in a cohort of 167 Rb patients between the years 2017 and 2023. We aim to establish genotype–phenotype correlations between the RB1 polymorphism and clinical presentations. By investigating these mutations in genetic testing, early detection of cancer and informed treatment decision‐making are possible. Ultimately, this will lead to precise genetic counseling, empowering parents to make informed choices about family planning and improving clinical management of both Rb patients and their predisposed relatives. Figure 3 provides an overview of the study from DNA extraction to investigating the correlation between RB1 mutations and clinical and outcome information. We hope that our preliminary findings are a promising step towards designing and implementing screening strategies in the future, with the potential to improve outcomes for retinoblastoma patients in Iran. However, further studies are necessary to confirm these results and investigate their clinical and therapeutic significance.

## Author Contributions

**Mohammad Faranoush:** conceptualization, investigation, writing – original draft, funding acquisition, methodology, validation, visualization, writing – review and editing, project administration, formal analysis, software, data curation, supervision, resources. **Masood Naseripour:** conceptualization, investigation, funding acquisition, writing – original draft, writing – review and editing, visualization; validation, methodology, software, formal analysis, project administration, resources, supervision, data curation. **Pooya Faranoush:** data curation, supervision, resources, project administration, formal analysis, software, methodology, validation, visualization, writing – original draft, funding acquisition, conceptualization, investigation, writing – review and editing. **Zeinab Davoodi‐Moghaddam:** conceptualization, investigation, funding acquisition, writing – original draft, methodology, validation, visualization, writing – review and editing, software, formal analysis, project administration, data curation, supervision, resources. **Alireza Jahandideh:** writing – original draft, writing – review and editing, supervision, data curation, software. **Negin Sadighnia:** conceptualization, investigation, funding acquisition, writing – original draft, writing – review and editing, visualization, validation, methodology, software, project administration, formal analysis, resources, supervision, data curation. **Delbar Daneshjou:** writing – original draft, conceptualization, methodology, visualization, writing – review and editing, project administration, software. **Parisa Shams:** software, project administration, writing – review and editing, writing – original draft, methodology, validation, data curation, conceptualization. **Ahad Sedaghat:** conceptualization, investigation, methodology validation, visualization, software, resources, data curation, writing – original draft. **Reza Mirshahi:** writing – original draft, conceptualization, funding acquisition, methodology, validation, software, resources, supervision, data curation. **Shirin Ravanbod:** conceptualization, funding acquisition, visualization, writing – original draft, writing – review and editing, project administration, resources, data curation, supervision, formal analysis, methodology. **Farzaneh Nasirnejad:** methodology conceptualization, writing – original draft, writing – review and editing, data curation, resources, project administration, software. **Ali Elahinia:** conceptualization, writing – original draft, writing – review and editing, visualization, methodology, investigation, resources, software. **Davood Bashash:** conceptualization, investigation, funding acquisition, writing – original draft, writing – review and editing, visualization, validation, methodology, software, formal analysis, project administration, resources, supervision, data curation.

## Ethics Statement

All the examinations were conducted with the ethical code of (IR.IUMS.REC.1398.1200) certified by the ethical committee of the Iran University of Medical Sciences.

## Conflicts of Interest

The authors declare no conflicts of interest.

## Supporting information


Figure S1.

## Data Availability

The data that support the findings of this study are available from the corresponding author upon reasonable request.
