# Supplementary material for: Delving Into Retinoblastoma Genetics: Discovery of Novel Mutations and Their Clinical Impact: Retrospective Cohort Study
Source: Cancer Med. 2025 May 2;14(9):e70922. doi: 10.1002/cam4.70922 (PMC12046630; doi:10.1002/cam4.70922)
Supplement: Supplementary file 1 — Figure S1. [file CAM4-14-e70922-s001.docx]

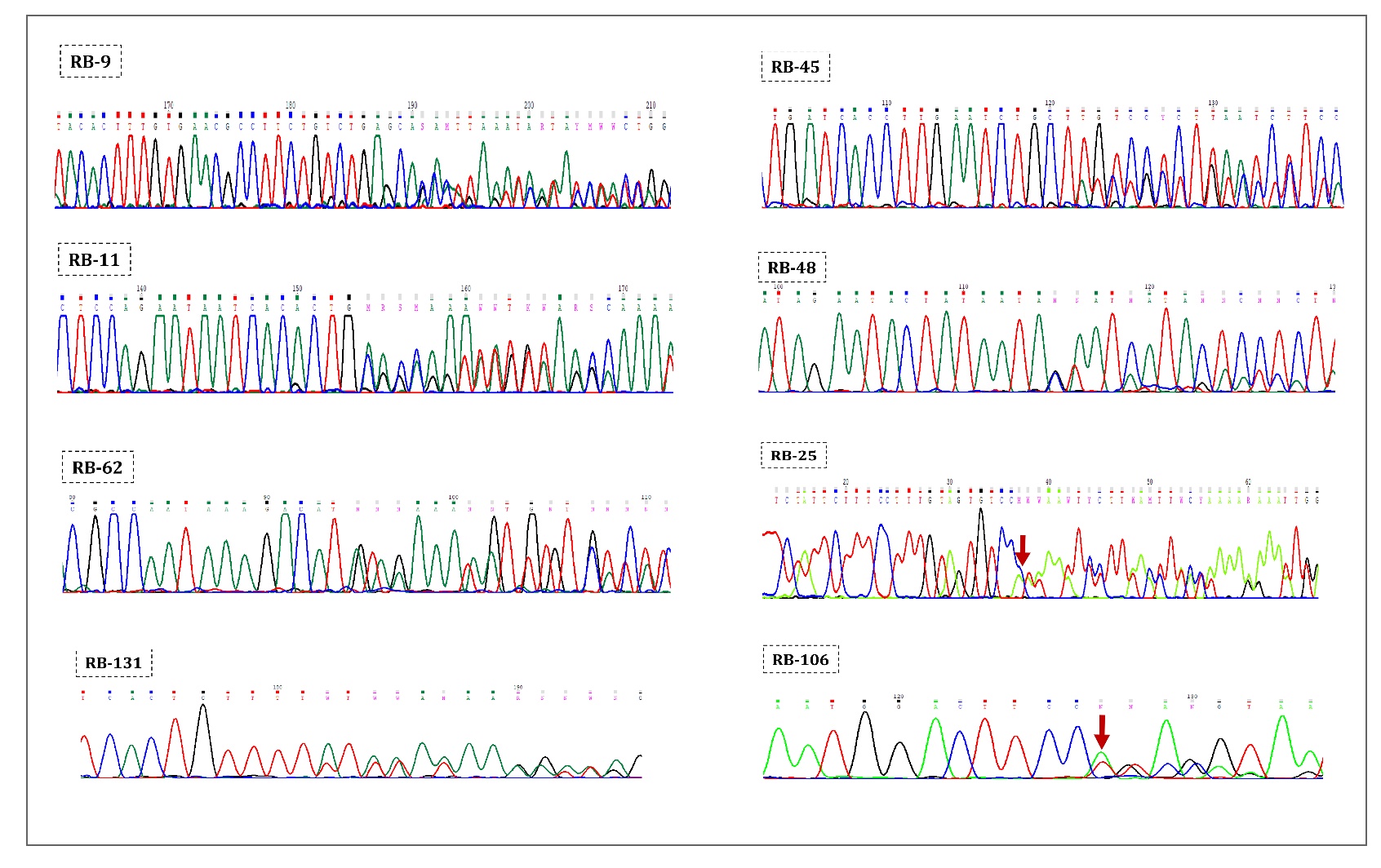


**Figure S1.** The sequencing analysis of the novel RB1 gene mutations (Without enucleation). ***RB-4 had exon deletion.**
